# A Systematic Pan-Cancer Analysis of YY1 Aberrations and their Relationship with Clinical Outcome, Tumor Microenvironment, and Therapeutic Targets

**DOI:** 10.1155/2022/5826741

**Published:** 2022-06-24

**Authors:** Xinghao Fu, Feihong Ji, Qi He, Xinguang Qiu

**Affiliations:** Department of Thyroid Surgery, First Affiliated Hospital of Zhengzhou University, Zhengzhou, Henan, China

## Abstract

Yin-Yang 1 (YY1) has a crucial function in the development of several malignancies, according to recent research. However, nothing is known about its aberrant expression and prognostic significance in human pan-cancer. We first explored the potential carcinogenic effect of YY1 in 33 cancers using the cancer genome atlas (TCGA) project and gene expression omnibus (GEO) datasets in this research. Then, we contained a variety of elements, for instance, gene expression, the state of survival, gene alterations, protein phosphorylation, immune infiltration, and related cellular pathways, and used a series of bioinformatics methods to investigate the underlying molecular mechanism of YY1 in the etiology or clinical prognosis of various malignancies. In most malignancies, YY1 was expressed at high levels, and the level of YY1 expression was statistically associated with the prognosis of tumor patients. The S118 site of YY1 implied higher phosphorylation expression in breast cancer, colon cancer, uterine corpus endometrial carcinoma (UCEC), and lung adenocarcinoma (LUAD) tumor tissues, but lower phosphorylation levels in ovarian cancer and clear cell carcinoma tumor tissues. For S247, higher phosphorylation levels were found in colon cancer, UCEC, and LUAD tumor tissue, and lower phosphorylation expression was found in clear cell carcinoma tumor tissue. In TCGA database, YY1 expression in BRCA, BRCA-LumA, BRCA-LumB, CESC, CHOL, COAD, ESCA, HNSC, HNSC-HPV-, KIRP, LGG, LIHC, and PAAD tumor tissues was a statistically significant positive connection of the estimated infiltration value of cancer-associated fibroblasts but a negative correlation in TGCT. In addition, the functional mechanism of YY1 also involves viral carcinogenesis and ribonucleic acid (RNA) metabolism related functions. Our first pan-cancer analysis offers a pretty comprehensive knowledge of YY1's oncogenic involvement in various cancers.

## 1. Introduction

Cancer is a leading cause of death and major obstacle affecting the quality of life in every country globally, and to date, the clinical outcome for most cancer patients remains poor [1, 2]. Lung, pancreas, and liver malignancies are the leading causes of cancer-related death worldwide [3–6]. The process of tumor genesis and development is extremely complex. Many genes could have a role in the occurrence of a certain cancer, and a certain gene may also have a role in the occurrence of various cancers. Therefore, it is critical to examine any significant genes' pan-cancer expression and to assess their relationship to clinical prognosis and probable molecular processes. In the research, we used high-throughput bioinformatics analysis to obtain data from the GEO database and the TCGA project for pan-cancer analysis [7–9].

The zinc finger transcription factor YY1 belongs to the Gli-Kruppel family [10]. YY1 expression is highly conserved from Xenopus to humans and regulates various downstream biological processes, such as cell proliferation, migration, cycle, and differentiation [11]. In tumor cells, YY1 plays a role in tumor angiogenesis by promoting hypoxia inducible factor 1 (HIF1)-dependent expression and vascular endothelial growth factor (VEGF) production [12–14], whereas the function of endothelium-specific YY1 in vascular development and angiogenesis is still unclear. Current researches have found that YY1 performs a cancer-promoting effect in breast [15], colon [16], stomach [17], and prostate cancer [18]. However, there is presently no indication of a pan-cancer connection between YY1 and many types of cancers based on extensive clinical data.

In this study, we aimed to aberrant expression of YY1 and its prognostic significance in human pan-cancer. For the pan-carcinogenesis investigation of YY1, we were the first to use the TCGA project and the GEO database. To inquire into the possible molecular processes of YY1 in the etiology or clinical prognosis of various malignancies, we considered a number of parameters such as gene expression, status of survival, gene alterations, protein phosphorylation, immunological infiltration, and associated cell pathways. Our findings suggested YY1 as a novel prognostic biomarker for many tumors and an immune therapy response indicator in most cancer types. Moreover, it may serve as a potential target for cancer therapy.

## 2. Materials and Methods

### 2.1. Analysis of Gene Expression

We used the website (http://timer.comp-genomics.org/) to analyze the expression differential of YY1 among cancer tissues in different tumors with nearby normal tissues in TCGA project. In the TCGA project, some neoplasms, including ACC and DLBC, do not have corresponding normal tissues. Therefore, we combined TCGA with GTEx database for analysis. Enter the website (http://gepia2.cancer-pku.cn/#analysis) to enter GEPIA2 [19], and type YY1 into the “Box Plot” gene search bar under the “Exploration Analysis” module to retrieve the gene. Set *P* value cutoff =0.01, log2FC (fold change) cutoff = 1, and “Match TCGA normal and GTEx data.” Furthermore, it is necessary to enter YY1 into the “Pathological Stage Plot” gene search bar under the “Exploration Analysis” module of GEPIA2 for retrieval. It was possible to create a violin diagram of YY1 expression in all TCGA tumors at various clinical phases (stage I, stage II, stage III, and stage IV). Set “log2(TPM+1) for log-scale” and select “Yes.”

Input website (http://ualcan.path.uab.edu/analysis-prot.html) into UALCAN; the site was a web tool used to analyze the cancer omics data, and we can use it to analyze protein expression in the CPTAC [20]. Enter “YY1” in the search box to search for the total protein or phosphorylated protein expression level among primary cancer and normal tissues. The CPTAC dataset contained data on six types of tumors, namely, LUAD, UCEC, colon cancer, breast cancer, ovarian cancer, and clear cell RCC.

Enter the website (https://www.proteinatlas.org/) into the Human Protein Atlas (HPA) database [21–23]; it is committed to providing all 24, 000 kinds of tissue and cell distribution data of human proteins. Enter “YY1” in the search box to search, and the RNA and protein expression of YY1 in various cancer cell lines, pathological tissues, and normal human tissues can be obtained.

### 2.2. Analysis of the Prognosis for Survival

Enter the website to enter GEPIA2, under “Survival Analysis” in the “Exploration Analysis” module, and click on “Survival Map.” YY1 was entered into the gene search bar to retrieve the OS and DFS data of YY1 in every TCGA cancers. The expression thresholds for separating the high-expression and low-expression cohorts were cutoff high (50%) and cutoff low (50%) values. The statistically significant tumors were then further visualized with survival maps.

The mRNA expression level and patients' clinical information of 414 BLCA tissues and 19 normal tissues, 539 KIRC tissues and 72 normal tissues, and 86 MESO tissues and 379 OV tissues were gotten from the TCGA database (https://portal.gdc.cancer.gov/). Clinical information about patients with BLCA included TNM stage, pathological stage, radiation therapy, gender, race, age, weight, height, BMI, histologic grade, subtype, lymphovascular invasion, smoking, survival time, and state of death. Clinical information of patients with KIRC includes TNM stage, pathological stage, gender, race, age, histologic grade, laterality, serum calcium, hemoglobin, survival time, and state of death. Clinical information of patients with MESO includes TNM stage, pathological stage, radiation therapy, gender, race, age, history asbestos exposure, laterality, time of survival, and state of death. Clinical information for patients with OV included FIGO stage, race, age, histologic grade, anatomic neoplasm subdivision, venous invasion, lymphatic invasion, tumor residual, tumor status, time of survival, and state of death. Univariate and multivariate Cox analyses were used to evaluate the relationship between YY1 gene and prognosis of patients, and the results were shown in tables and forest maps. The median expression value of YY1 was considered to be the cutoff value.

### 2.3. Analysis of Genetic Alteration

Input website (https://www.cbioportal.org/) into cBioPortal, under “TCGA Pan Cancer Atlas Studies” in the “Query” module, click on “Query by Gene”, and enter YY1 in the gene search bar to retrieve the genetic variation characteristics of YY1 [24, 25]. You may see the mutation frequency, mutation site, and copy number change of genes in all TCGA tumors by going to the “Cancer Types Summary” section. If you go to the “Mutations” module, you can see the YY1 mutation site information, and then, click “View 3D Structure”; you can display the 3D structure diagram of YY1 gene. Then, we used the “Comparison/Survival” module to produce Kaplan-Meier plots and gather data on total OS, DFS, progression-free survival (PFS), and DSS differences of TCGA cancer cases with and without YY1 mutation.

Return to the home page of cBioPortal, input all tumor names of TCGA in the search bar under “Query” module, select the data from the “TCGA Pan Cancer Atlas” project, click “Query by Gene,” and input YY1 in the gene search bar for search. The effect of YY1 mutation on expression in all TCGA tumors could be observed by clicking the “mRNA vs mut type” under the “Plots” module.

Enter the website (http://www.mutarget.com/) to access the muTarget database [26]. Enter the “Genotype” and “Target” module, respectively, all tumor types were selected, and the rest were set as default Settings. YY1 was input in the gene search bar for retrieval, and it could be observed which gene expression was affected by the mutation of YY1 in different tumors and which gene mutation had an impact on the expression of YY1 in different tumors.

### 2.4. Immune Infiltration Analysis

Input website (http://timer.comp-genomics.org/) into the TIMER2, enter YY1 in the “Gene Expression” functional bar under the “Immune” module, and select “Cancer associated fibroblast” in the functional bar of “Immune Infiltrates” for search, to observe and analyze the association among the expression of YY1 in various tumors of TCGA project and immune infiltration. Immune infiltration was estimated using the XCELL, MCPCOUNTER, and EPIC methods. Purity adjustment Spearman's rank correlation test was utilized to determine the *P* values and partial correlation (COR) values. Heat maps and scatter maps are used to illustrate the data.

The high-throughput sequencing RNA data [fragments per kilobase per million (FPKM) format] of 510 THCA tissues and 58 normal tissues, 414 BLCA tissues and 19 normal tissues, 539 KIRC tissues and 72 normal tissues, 379 OV tissues, 79 ACC tissues, 1109 BRCA tissues and 113 normal tissues, 306 CESC tissues and 3 normal tissues, 289 KIRP tissues and 32 normal tissues, 36 CHOL tissues and 9 normal tissues, 480 COAD tissues and 41 normal tissues, 48 DLBC tissues, 162 ESCA tissues and 11 normal tissues, 169 GBM tissues and 5 normal tissues, 502 HNSC tissues and 44 normal tissues, 62 kidney chromophobe (KICH) tissues and 24 normal tissues, 151 LAML tissues, 529 LGG tissues, 374 LIHC tissues and 50 normal tissues, 535 LUAD tissues and 59 normal tissues, 502 LUSC tissues and 49 normal tissues, 86 MESO tissues, 178 PAAD tissues and 4 normal tissues, 183 pheochromocytoma and paraganglioma (PCPG) tissues and 3 normal tissues, 499 prostate adenocarcinoma (PRAD) tissues and 52 normal tissues, 167 rectum adenocarcinoma (READ) tissues and 10 normal tissues, 263 sarcoma (SARC) tissues and 2 normal tissues, 471 SKCM tissues and 1 normal tissues, 375 STAD tissues and 32 normal tissues, 183 TGCT tissues, 119 THYM tissues and 2 normal tissues, 552 UCEC tissues and 35 normal tissues, 56 uterine carcinosarcoma (UCS) tissues, 80 uveal melanoma (UVM) tissues, 329 oral squamous cell carcinomas (OSCC) tissues and 32 normal tissues, 80 esophageal adenocarcinoma (ESAD) tissues and 10 normal tissues, 82 esophageal squamous cell carcinoma (ESCC) tissues, and 1 normal tissue were gotten from the TCGA database (https://portal.gdc.cancer.gov/). First, we used the single sample GSEA method from the R package “GSVA” [27] to present infiltration enrichment of 24 common immune cells, including DCs, immature DCs (iDCs), activated DCs (aDCs), plasmacytoid DCs (pDCs), T cells, T helper (Th) cells, type 1 Th cells (Th1), Th2, type 17 Th cells (Th17), regulatory T cells (Treg), T gamma delta (Tgd), Tcm, T effector memory (Tem), T follicular helper (Tfh), CD8^+^ T cells, B cells, neutrophils, macrophages, cytotoxic cells, mast cells, eosinophils, NK cells, NK 56^−^ cells, and NK 56^+^ cells. Next, the association between YY1 expression and immune cell infiltration was evaluated by Spearman's analysis. The results are presented in tables and lollipop charts.

### 2.5. Analysis of YY1-Related Gene Enrichment

Enter the URL (https://string-db.org/) to enter the STRING website, enter YY1 in the “Protein Name” function bar, and select “Homo sapiens” in the “Organism” function bar to search for it. Then, set the following conditions in the settings options: minimum required interaction score [“Low confidence (0.150)”], meaning of network edges (“evidence”), max number of interactors to show (“no more than 50 interactors” in 1st shell), and active interaction sources (“experiments”) submit the result to get YY1-binding protein.

We used GEPIA2 to find the first 100 target genes linked to YY1. Subsequently, several genes were selected from the 100 target genes, and using the “Correlation Analysis” function of GEPIA2, Pearson correlation analysis was done between YY1 and the chosen genes. For the dot plot, the log2 TPM was used. The correlation coefficient (*R*) and the *P* value were shown. Finally, a heat map of the selected genes was created using the TIMER2 website's “Gene_Corr” feature.

Input website (http://bioinformatics.psb.ugent.be/webtools/Venn/) into the VENN website, and combine the YY1-related proteins and the first 100 target genes linked to YY1 cross analysis [28]. In addition, we conducted KEGG pathway analysis and GO enrichment analysis by combining the two sets of data. In short, we upload the list of genes to DAVID, and set the selected identifier (“OFFICIAL_GENE_SYMBOL”) and species (“Homo sapiens”) to get the functional annotation table data. The “clusterProfiler” and “ggplot2” R package was then used to visualize the enriched pathways [29].

According to the median value of YY1 expression in different tumor samples in the collected TCGA dataset, the gene expression data were divided into high-expression group and low-expression group. GSEA analysis software [30] was used to analyze the signal pathway enrichment of YY1 using the c2.cp.v7.2.symbols.gmt dataset of molecular signature database (MsigD) as the functional gene set. Take the absolute value of NES ≥2.0, *P* value <0.05; FDR *q* value <0.25 was used to screen the enrichment results of YY1 single gene.

### 2.6. Statistical Analyses

All statistical analyses were conducted using R (version 3.6.0). *P* < 0.05 was considered statistically significant.

## 3. Results

### 3.1. Analysis Data of Gene Expression

**Figure 1:**
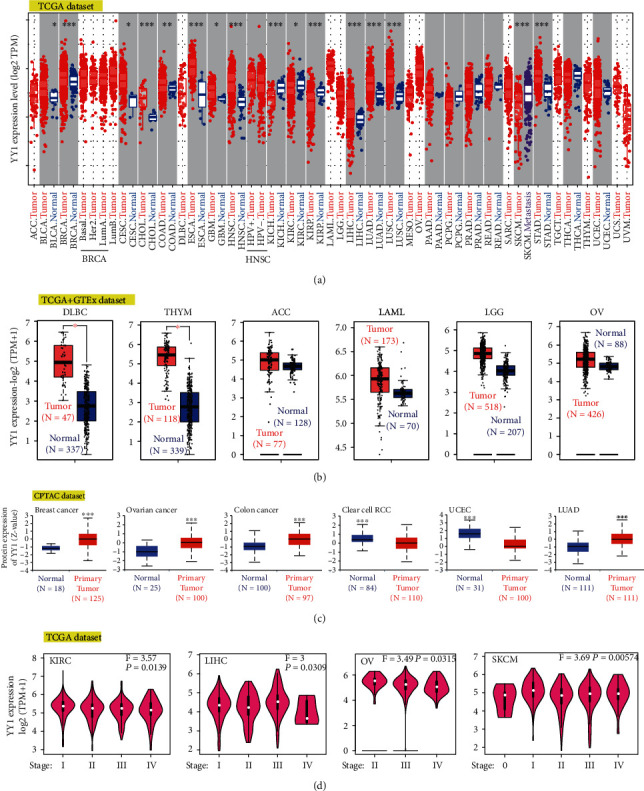
YY1 gene expression levels in various tumors and pathological stages. (a) The expression level of YY1 gene in various malignancies was investigated by TCGA dataset. (b) The matching normal tissues from the GTEx database were employed as controls for the types of DLBC, THYM, ACC, LAML, LGG, and OV in the TCGA project. The data for the box plot was provided. (c) We compared the expression levels of total YY1 protein among normal tissues and primary tissues of breast cancer, ovarian cancer, colon cancer, clear cell RCC, UCEC, and LUAD based on the CPTAC dataset. (d) According to TCGA dataset, the degree of expressiveness of YY1 gene was analyzed according to the major pathological stages (stage I, II, III, and IV) of KIRC, LIHC, OV, and SKCM. Log-scale uses Log2 (TPM+1). ∗*P* < 0.05; ∗∗*P* < 0.01; and ∗∗∗*P* < 0.001.

The goal of this research was to investigate the carcinogenic effect of human YY1 (mRNA localization at NM_003403.5, protein localization at NP_003394.1). The TCGA database was utilized to evaluate the expression of YY1 in various kinds of tumors using tumor immune estimation resource, version 2 (TIMER2). As shown in Figure 1(a), the expression levels of YY1 in the tumor tissues of bladder urothelial carcinoma (BLCA), BRCA, CHOL, ESCA, HNSC, LIHC, LUAD, lung squamous cell carcinoma (LUSC), stomach adenocarcinoma (STAD) (*P* < 0.001), COAD (*P* < 0.01), CESC, and glioblastoma multiforme (GBM) (*P* < 0.05) were higher than the corresponding control tissues.

We evaluated the difference in YY1 expression among normal and tumor tissues of lymphoid neoplasm diffuse large B-cell lymphoma (DLBC) and thymoma (THYM) using normal tissues from the genotype-tissue expression (GTEx) dataset as controls (Figure 1(b), *P* < 0.01). Nonetheless, the data did not differ significantly for other tumors, such as adrenocortical carcinoma (ACC), acute myeloid leukemia (LAML), LGG, or ovarian serous cystadenocarcinoma (OV).

When compared to normal tissues, YY1 total protein expression was higher in the primary tissues of breast, ovarian, colon, and lung adenocarcinoma and lower in the primary tissues of clear cell renal cell carcinoma (clear cell RCC) and UCEC (Figure 1(c), *P* < 0.001).

Then, we utilized gene expression profiling interactive analysis, Version 2 (GEPIA2)'s pathological staging mapping module to investigate the association among YY1 expression and cancer pathological staging, and the results showed statistical significance in kidney renal clear cell carcinoma (KIRC), LIHC, OV, and skin cutaneous melanoma (SKCM) (Figure 1(d), all *P* < 0.05), but no statistical significance in other cancers.

**Figure 2:**
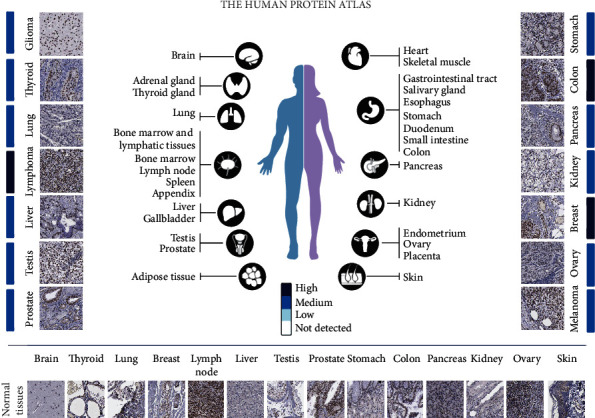
YY1 is highly expressed in many types of tumors. Immunohistochemistry was performed with rabbit polyclonal antibody HPA001119 (Sigma Aldrich).

Subsequently, we used HPA database to analyze the RNA and protein expressions of YY1 in various cancer cell lines, pathological tissues, and normal human tissues. As shown in supplementary Figure S1, at the RNA level, YY1 is highest in bone marrow, followed by the thymus and liver. At the protein level, YY1 was highly expressed in urinary bladder, testis, ovary, and placenta tissues (Supplementary Figure S2). Among various cancer cell lines, U-698 and MOLT-4 cell lines ranked first and second in YY1 expression levels, which were lymphoid derived malignancies, followed by HL-60 and THP-1 cell lines, which were bone marrow derived (Supplementary Figure S3). Among various human cancer tissues, protein expression of YY1 was highest in head and neck tumors, followed by breast cancer (Supplementary Figure S4). Finally, immunohistochemical analysis showed higher YY1 levels in significant proportions of breast cancer, colorectal cancer, and lymphoma (Figure 2).

### 3.2. Survival Analysis Data

**Figure 3:**
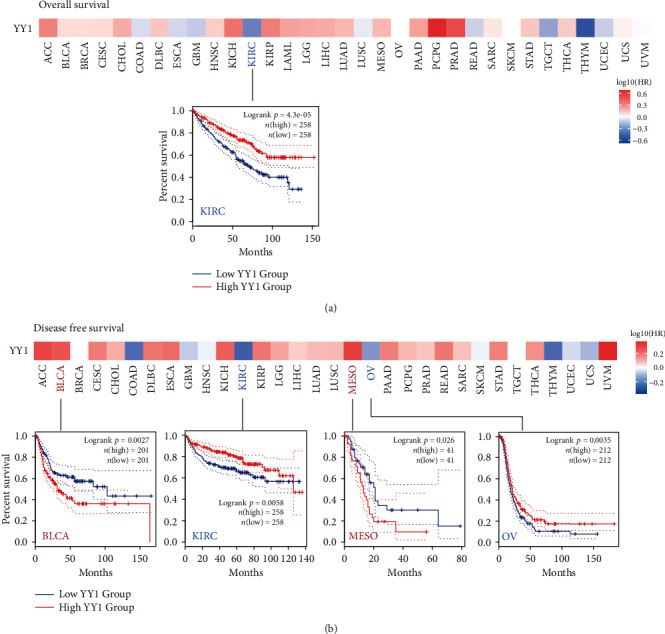
Association between YY1 gene expression and tumor survival prognosis in TCGA. By analyzing the YY1 expression of different cancers in the TCGA using the GEPIA2, we were able to determine the OS (a) and DFS (b). With remarkable results, a survival diagram and Kaplan-Meier curve were shown.

Tumor cases were separated into high-expression and low-expression groups based on YY1 expression levels, and then, the TCGA and GEO datasets were utilized to investigate the link among YY1 expression and the prognosis of patients with various malignancies. As shown in Figure 3(a), low expression of YY1 in the TCGA database was associated with poor overall survival (OS) of KIRC (*P* < 0.001). High expression of YY1 was related with poor prognosis of BLCA (*P* = 0.0027) and mesothelioma (MESO) (*P* = 0.026) in TCGA patients, according to disease free survival (DFS) study data (Figure 3(b)). In addition, low expression of YY1 gene was linked to poor DFS of KIRC (*P* = 0.0058) and OV (*P* = 0.035). The results showed that YY1 expression differed from the prognosis of various cancers.

**Figure 4:**
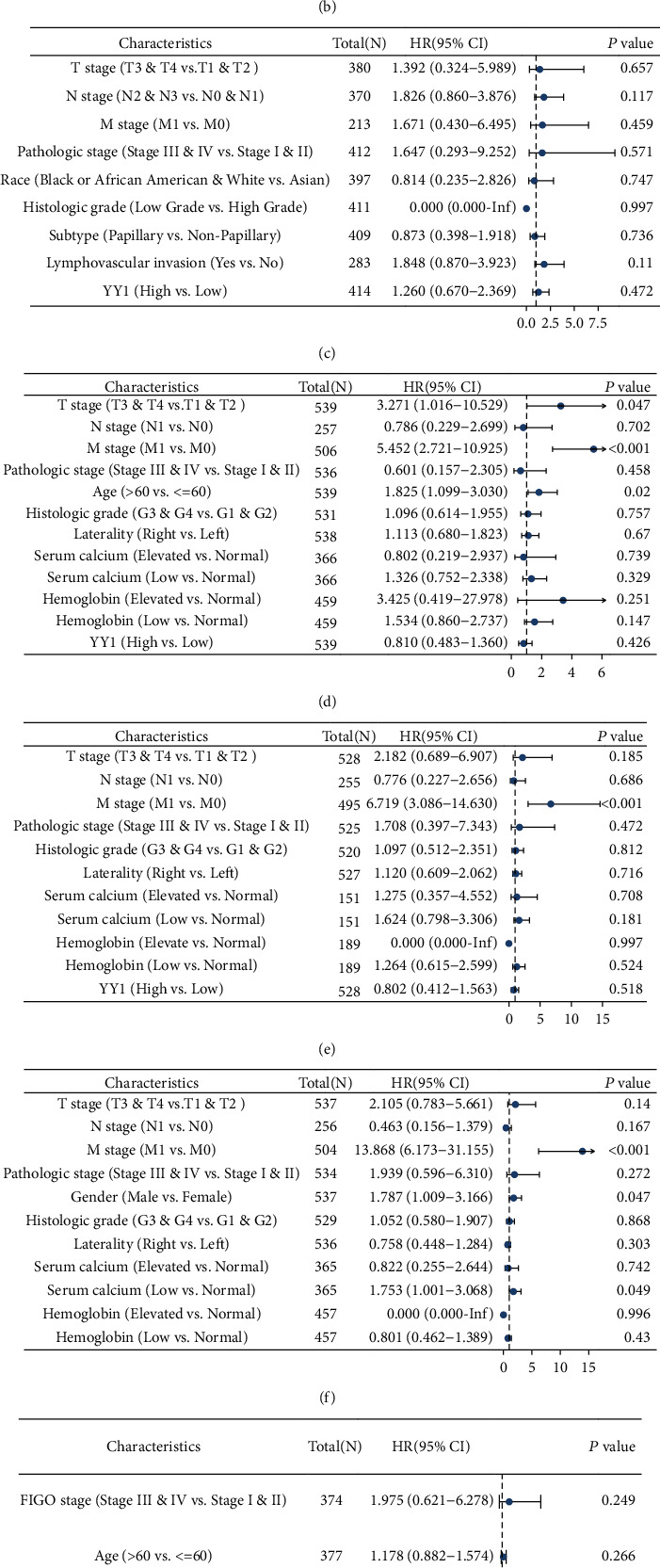
Relationships between clinicopathologic characteristics and survival outcomes of BLCA, KIRC, and OV patient through multivariate Cox regression analysis. (a) Relationships between clinicopathologic factors and OS of BLCA patients by the use of multivariate assays. (b) Relationships between clinicopathologic factors and DSS of BLCA patients by the use of multivariate assays. (c) Relationships between clinicopathologic factors and PFI of BLCA patients by the use of multivariate assays. (d) Relationships between clinicopathologic factors and OS of KIRC patients by the use of multivariate assays. (e) Relationships between clinicopathologic factors and DSS of KIRC patients through by the use of multivariate assays. (f) Relationships between clinicopathologic factors and PFI of KIRC patients by the use of multivariate assays. (g) Relationships between clinicopathologic factors and OS of OV patients by the use of multivariate assays. (h) Relationships between clinicopathologic factors and PFI of OV patients through by the use of multivariate assays.

Univariate Cox analysis showed that high expression of YY1 was correlated with poor progress free interval (PFI)of BLCA, while low expression of YY1 was correlated with poor OS and poor disease-specific survival (DSS) of KIRC and poor PFI of OV (Supplementary Tables S1–S12). Multivariate Cox analysis showed that YY1 was not an independent prognostic factor of BLCA, KIRC, MESO, and OV (Figures 4(a)–4(h)).

### 3.3. Analysis Data of Genetic Alteration

**Figure 5:**
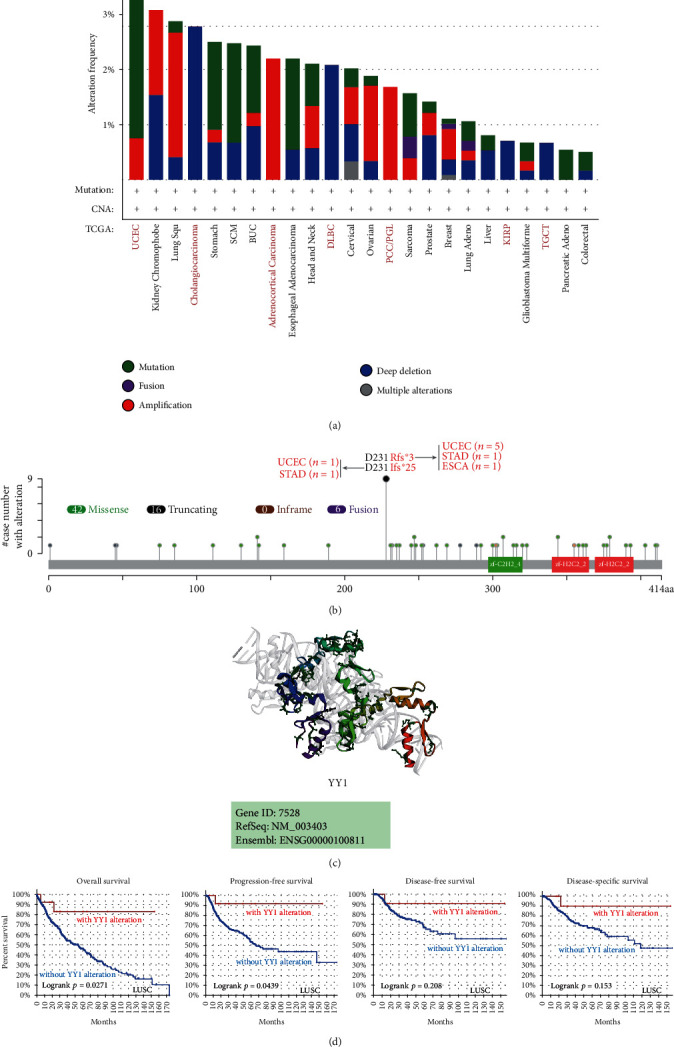
Mutation characteristics of YY1 in various TCGA cancers. The features of YY1 mutations in TCGA cancers were examined using the cBioPortal. The frequency of alteration with mutation type (a) and site (b) is shown. The 3D structure of YY1 is displayed (c). We also utilized the cBioPortal to explore the possible association between mutation status and OS, DSS, DFS, and PFS of LUSC (d).

In the TCGA projects, we looked at the genetic alteration status of the YY1 gene in various tumor samples. As demonstrated in Figure 5(a), sufferers with UCEC with “mutation” as the main form had the greatest YY1 alteration frequency (>3%). In adrenocortical carcinoma and pheochromocytoma/paraganglioma (PCC/PGL) instances, the “amplification” type of copy number alteration (CNA) was the only kind seen, with a frequency of 2.2 percent and 1.69 percent, respectively (Figure 5(a)). It is important to note that all cholangiocarcinoma, DLBC, and KIRP cases with genetic alteration had copy number deletion of YY1 (Figure 5(a)).  Figure 5(b) shows the types, loci, and number of instances with YY1 genetic mutation. We discovered that the most common sort of genetic mutation was a YY1 missense mutation, while the D231Rfs∗3 and D231Ifs∗25 mutations at the D231 site were detected in 5 cases of UCEC, 1 case of STAD, 1 case of ESCA, 1 case of UCEC, and 1 case of STAD, respectively (Figure 5(b)), which could induce the frame shift mutation of YY1 gene, translation from Aspartic acid (D) to Arginine (R) or Isoleucine (I) at YY1 protein 231 and subsequent truncation of YY1 protein, respectively. The 3D structure of the YY1 protein can be seen (Figure 5(c)). We also looked at the possibility of a link between a YY1 gene mutation and clinical survival prognosis in individuals with various kinds of tumor. The data in Figure 5(d) show that LUSC patients with YY1 mutation had better overall prognosis (*P* = 0.0271) and progression-free (*P* = 0.0439) survival, but there was no significant correlation between DFS (*P* = 0.208) and DSS (*P* = 0.153) compared to individuals who do not have the YY1 mutation.

**Figure 6:**
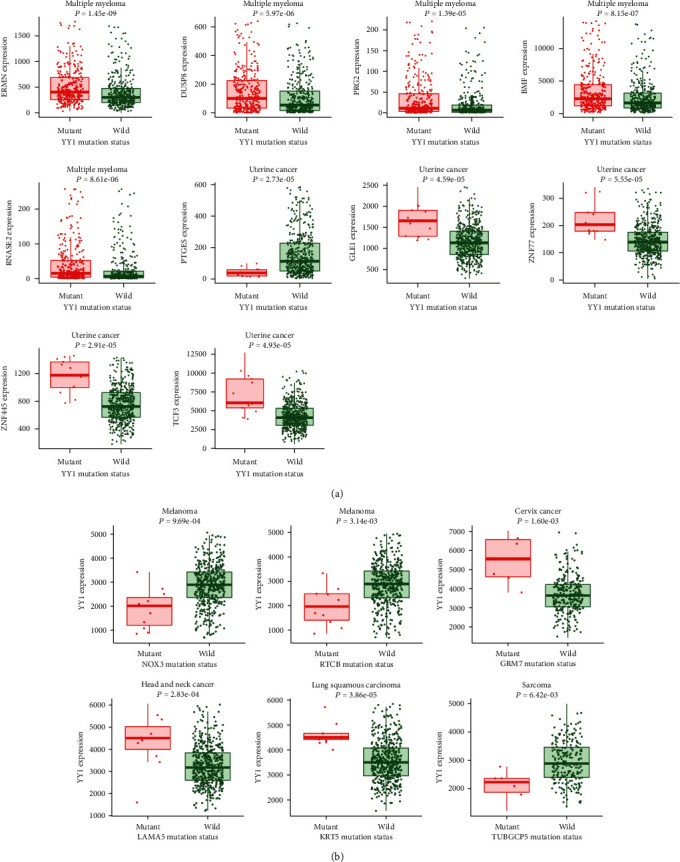
The effect of YY1 mutation on gene expression and gene mutation on YY1 expression in various cancers. (a) The effect of YY1 mutation on gene expression in multiple myeloma and uterine cancer. (b) The effect of gene mutation on YY1 expression in cervix cancer, head and neck cancer, lung squamous cell carcinoma, sarcoma, and melanoma.

We observed the effects of YY1 mutations in different tumor samples in the TCGA cohort on gene expression. Due to the small number of tumor samples with YY1 gene mutation in the database, all data had no statistical significance (Supplementary Figure S5). Then, we used muTarget database to observe which gene expression was affected by the mutation of YY1 in different tumors and which gene mutation had an impact on the expression of YY1 in different tumors. As shown in Figure 6(a), YY1 gene mutation can affect the expression of ermin (ERMN), dual specificity phosphatase 8 (DUSP8), proteoglycan 2 (PRG2), bcl2 modifying factor (BMF), ribonuclease RNase A family 2 (RNASE2) genes in multiple myeloma and prostaglandin E synthase (PTGES), GLE1 RNA export mediator (GLE1), zinc finger protein 77 (ZNF77), zinc finger protein 445 (ZNF445), and transcription factor 3 (TCF3) genes in uterine cancer. In cervix cancer, the mutation of glutamate receptor, metabotropic 7 (GRM7) gene can increase the expression of YY1 (Figure 6(b)). In head and neck cancer, the mutation of laminin alpha 5 (LAMA5) gene can increase the expression of YY1 (Figure 6(b)). In lung squamous cell carcinoma, keratin 5 type (IIKRT5) gene mutation can increase the expression of YY1 (Figure 6(b)). In sarcoma, the mutation of tubulin gamma complex-associated protein 5 (TUBGCP5) gene reduced the expression of YY1 (Figure 6(b)). In melanoma, NADPH oxidase 3 (NOX3) and RNA 2',3'-cyclic phosphate and 5'-OH ligase (RTCB) mutations reduce YY1 expression (Figure 6(b)).

### 3.4. Analysis Data of Protein Phosphorylation

**Figure 7:**
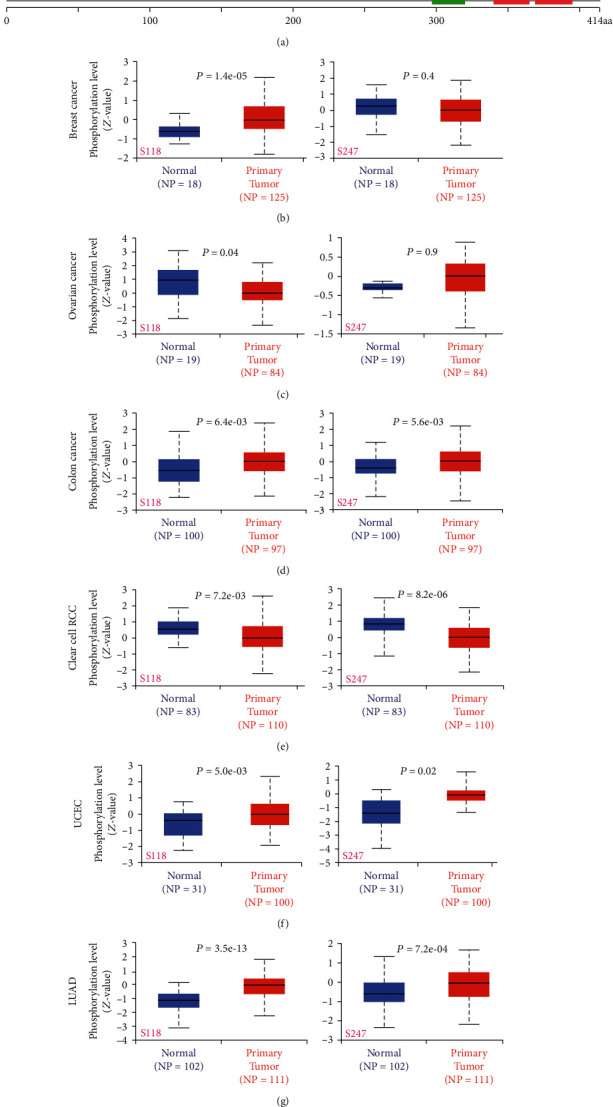
Phosphorylation of the YY1 protein in various tumors. We used UALCAN to compare the expression levels of YY1 phosphorylated proteins (NP_003394.1, S118, and S247 sites) among chosen normal tissue and primary tissue using the CPTAC. The phosphoprotein locations that yielded positive findings are depicted in the YY1 protein's schematic design (a). We provide the box plots for various malignancies such as breast cancer (b), ovarian cancer (c), colon cancer (d), clear cell RCC (e), UCEC (f), and LUAD (g).

We also analyzed how phosphorylation levels of YY1 differed among normal and tumor tissues. The phosphorylation sites of YY1 and their substantial changes are summarized in Figure 7(a). Six tumor types (breast cancer, ovarian cancer, colon cancer, clear cell RCC, UCEC, and LUAD) were studied based on the clinical proteomic tumor analysis consortium (CPTAC) dataset (Figures 7(b)–7(g)). The S118 site of YY1 showed higher phosphorylation levels in breast cancer, colon cancer, UCEC, and LUAD tumor tissues (all *P* < 0.05) and showed lower phosphorylation levels in ovarian and clear cell carcinomas (both *P* < 0.05). For S247, higher phosphorylation levels were observed in colon cancer, UCEC, and LUAD tumors (all *P* < 0.05), and clear cell carcinoma showed lower phosphorylation levels in tumor tissues (*P* < 0.05). This observation provided a deeper molecular analysis method to further explore the potential function of YY1 phosphorylation in the occurrence and development of tumors.

### 3.5. Immune Infiltration Analysis Data

**Figure 8:**
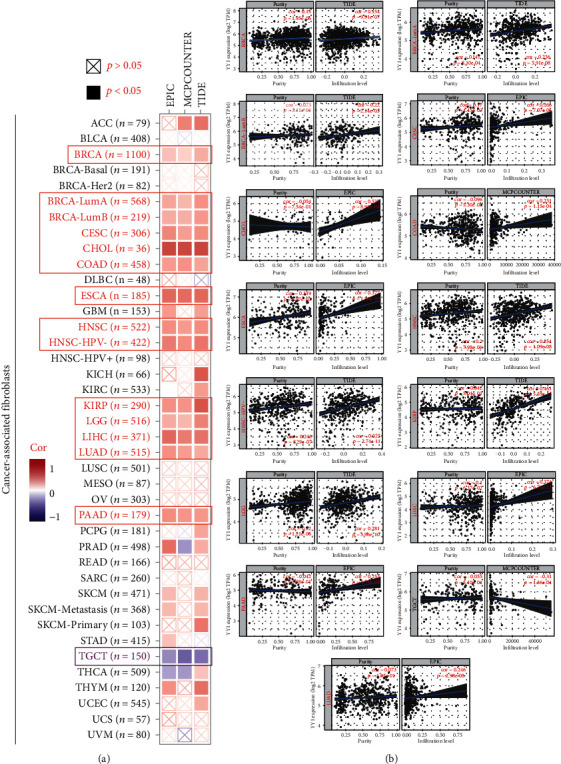
The relationship between immune infiltration of tumor-related fibroblasts and YY1 expression. The possible association between YY1 gene expression level in TCGA and tumor-associated fibroblast infiltration level was investigated using several methods.

Tumor-infiltrating immune cells were intimately linked to the occurrence, growth, and metastasis of malignancies as an essential component of the tumor microenvironment. The activity of a number of tumor-infiltrating immune cells has been reported to be regulated by tumor-associated fibroblasts in the stroma of the tumor microenvironment. We discovered that YY1 expression in BRCA, BRCA-LumA, BRCA-LumB, CESC, CHOL, COAD, ESCA, HNSC, HNSC-HPV-, KIRP, LGG, LIHC, LUAD, and PAAD tumor tissues had a statistically positive correlation with the estimated infiltration value of cancer-associated fibroblasts in the TCGA database, but had a statistically negative correlation in TGCT (Figures 8(a) and 8(b)).  Figure 8 shows the scatterplot data of the aforesaid tumors generated using one algorithm. According to the MCPCOUNTER method, the amount of YY1 expression in TGCT was negatively associated with the level of invasion of cancer-related fibroblast (Figures 8(a) and 8(b), cor = −0.31, *P* = 1.36e − 04).

**Figure 9:**
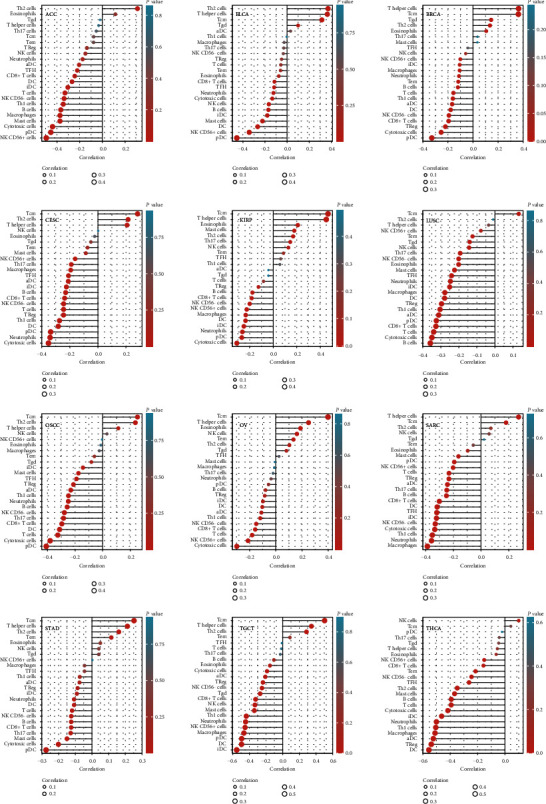
The association between YY1 expression and tumor-infiltrating immune cells in various cancers.

We first detected the 24 immune cell types in 36 tumor types from TCGA database by ssGSEA method and then investigated the relationship between YY1 and immune cell infiltration by Spearman's analysis. We selected 16 types of cancer data, among which YY1 expression level was most closely related to a variety of tumor-infiltrating immune cells. These data were presented in the form of a lollipop chart (Figure 9). As shown in Figure 9, T central memory (Tcm) is negatively correlated with YY1 expression in ACC, but significantly positively correlated with YY1 expression in the remaining 15 tumors. Type 2 Th cells (Th2) cells showed negative correlation with YY1 expression in thyroid carcinoma (THCA), no correlation with YY1 expression in LUSC, and positive correlation with YY1 expression in the remaining 14 tumors. T helper cells were not associated with YY1 expression in ACC, LUSC, and THCA, but positively correlated with YY1 expression in the remaining 13 tumors. In ACC, YY1 expression was positively correlated with Th2 cells and eosinophils, but negatively correlated with other immune cells. In LUSC, YY1 expression was positively correlated with Tcm but negatively correlated with other immune cells. In THCA, YY1 expression was positively correlated with natural killer (NK) cells and Tcm, but negatively correlated with other immune cells.

### 3.6. Enrichment Analysis of YY1-Related Partners

**Figure 10:**
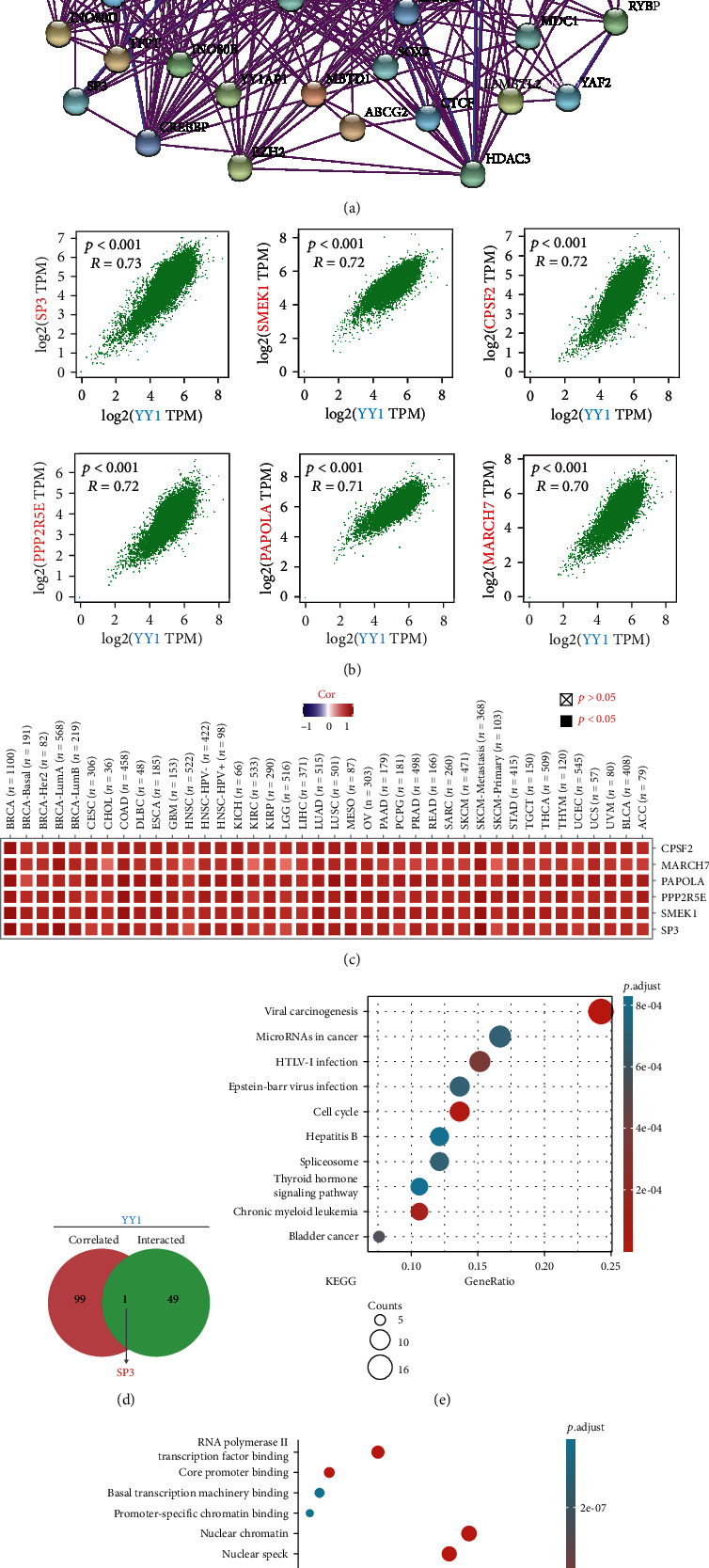
Enrichment analysis of YY1 related genes. (a) Using the STRING tool, we first gathered experimentally determined YY1-binding proteins. (b) The top 100 YY1 associated genes in the TCGA project were obtained using the GEPIA2 method, and the expression association between YY1 and SP3, SMEK1, CPSF2, PPP2R5E, PAPOLA, MARCH7, and other selected target genes was analyzed. (c) Display the corresponding heat map data for detailed cancer types. (d) YY1-binding and related genes were cross-analyzed. (e) YY1 binding and interaction genes were used to create a KEGG pathway analysis. (f) YY1 binding and interaction genes were used to create a GO enrichment analysis.

We attempted to filter out the targeted binding protein of YY1 and YY1 expression-associated genes for a suite of pathway enrichment analyses in order to better understand the underlying mechanism of YY1 gene in carcinogenesis. We found 50 YY1-binding proteins based on the STRING program, all of which are validated by experimental data.  Figure 10(a) depicts the result. The first 100 genes linked with YY1 expression were discovered using the GEPIA2 algorithm and TCGA tumor expression data. As illustrated in Figure 10(b), YY1 expression level is positively associated with SP3 transcription factor (SP3) (*R* = 0.73), SMEK homolog 1, suppressor of MEK1 (SMEK1) (*R* = 0.72), cleavage and polyadenylation-specific factor 2 (CPSF2) (*R* = 0.72), protein phosphatase 2regulatory subunit B'epsilon (PPP2R5E) (*R* = 0.72), Poly(A) polymerase alpha (PAPOLA) (*R* = 0.71), and membrane-associated ring finger(C3HC4)7 (MARCH7) (*R* = 0.70) genes (*P* < 0.001). The heat map data revealed a positive association between YY1 and the top five genes in many cancers (Figure 10(c)). The two groups were cross-analyzed, and one common molecule was obtained, namely SP3 (Figure 10(d)).

For Kyoto Encyclopedia of Genes and Genomes (KEGG) and Gene Ontology (GO) enrichment analysis, we merged these two datasets. According to the KEGG data in Figure 10(e), YY1's function in tumor pathogenesis may be linked to “viral carcinogenesis.” The majority of these genes was also related with “covalent chromatin modification” or “histone modification” according to GO enrichment analysis results (Figure 10(f)).

**Figure 11:**
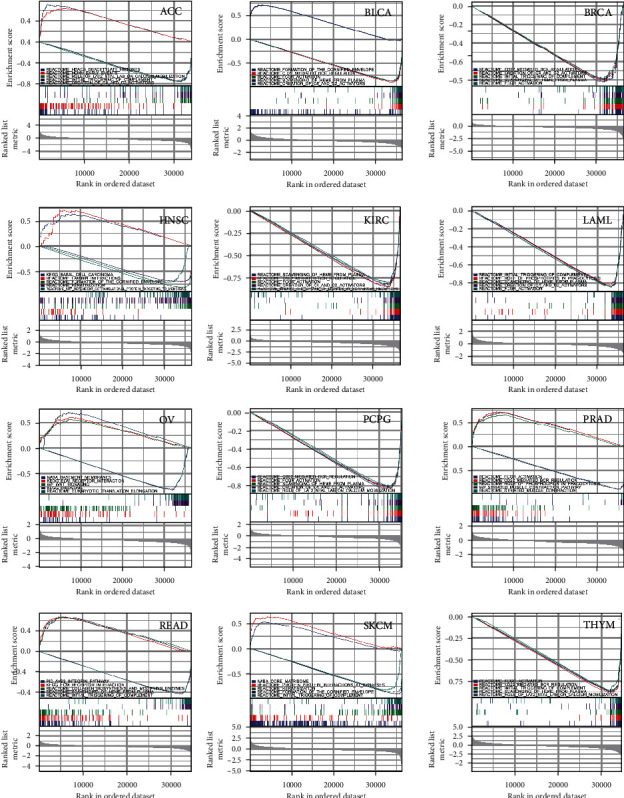
GSEA analysis of the effect of YY1 expression on cell signaling pathway in various cancers.

GSEA analysis was performed to explore the signaling pathways affected by YY1 expression in 36 tumors. Among the results, none of the enrichment results of CHOL, COAD, DLBC, KICH, LUAD, STAD, and ESAD met *P* value < 0.05 and FDR *q* value < 0.25 condition. GSEA analysis results of ACC, BLCA, BRCA, HNSC, KIRC, LAML, OV, PCPG, PRAD, READ, SKCM and THYM are shown in Figure 11. For example, GSEA analysis results show that YY1 expression in OV may interact with basement membranes, extracellular matrix (ECM) receptor interaction, Wnt signaling, and ribosome and eukaryotic translation of plants (Figure 11).

## 4. Discussion

In many cancer types, the expression of YY1 in tumors was considerably greater than in normal tissues, indicating that YY1 may play a part in tumor incidence and progression, whereas whether YY1 plays a part in the occurrence and development of many cancers via common molecular pathways is unknown. By searching relevant literature, we could not find relevant articles about YY1 pan-cancer analysis. Hence, by using the data from TCGA, CPTAC, and GEO, we performed a pan-cancer analysis of YY1 gene in a total of 33 different tumors, and the results suggested that the mechanism of YY1 action was different in various tumors. In this work, we analyzed the function of YY1 in various malignancies from a macro viewpoint in order to get a better knowledge of cancer etiology and enhance cancer detection and therapy.

Previous researches have found that YY1 was associated with the development of many malignancies. A large number of experiments have proved that YY1 was highly expressed in many tumors, including breast cancer [31], prostate cancer [32], colon cancer [33], ovarian cancer [34], esophageal cancer [35], nervous system tumors [36], pancreatic cancer [37], osteosarcoma [38], and melanoma [39]. Qu et al. recently showed that by directly inhibiting c-MYC target of laryngeal cancer cells (MYCT1), YY1 promoted the proliferation and migration of laryngeal cancer cells while preventing apoptosis. The authors also discovered that in patients with metastatic laryngeal cancer, YY1 levels were greater, and MYCT1 levels were lower than in individuals without metastasis. These data imply that YY1 might be a potential laryngeal cancer target [40]. Gabriela et al. found that in acute lymphoblastic leukemia (ALL), YY1 overexpression was associated with significantly reduced survival rate [41]. Zhao et al. discovered that the expression of YY1 in cancer tissues of melanoma patients was higher than in benign nevus and normal tissue control groups and that silencing of YY1 may inhibit melanoma cells from proliferating, migrating, and invading. Increased YY1 levels were linked to tumor metastasis and stage [39]. We should be aware of the influence of elevated YY1 expression on the diagnosis and treatment of these cancers and utilize this as a guide to enhance the diagnosis and treatment of associated tumors.

Although YY1 was overexpressed in many cancers, the survival and prognosis analysis results of YY1 gene suggested that different conclusions will be drawn in different tumors. In BLCA and MESO, YY1 overexpression was closely connected with poor prognosis. In the clinical work of these tumors, attention should be paid to the effect of increased YY1 expression, which can guide the diagnosis and treatment of related tumors. In KIRC and OV, low YY1 expression has also been linked to a bad prognosis, suggesting that the functions of YY1 are diversified and further experiments are needed to verify them.

In this study, TCGA database was analyzed by cBioPortal; sufferers with UCEC with “mutation” as the main form had the greatest YY1 alteration frequency (>3%). In adrenocortical carcinoma and PCC/PGL instances, the “amplification” type of CNA was the only kind seen, with a frequency of 2.2 percent and 1.69 percent, respectively. It is important to note that all cholangiocarcinoma, DLBC, and KIRP cases with genetic alteration had copy number deletion of YY1. The most common sort of genetic mutation was a YY1 missense mutation. Abnormal changes in gene level are one of the important reasons for abnormal expression of YY1, and patients with high mutation rate of YY1 have a relatively good prognosis. Moreover, we found that YY1 mutation could affect the expression changes of certain genes in some cancers; for example, in multiple myeloma, YY1 mutation could affect the expressions of ERMN, DUSP8, PRG2, BMF, and RNASE2 genes. And in some cancers, the expression of YY1 is affected by mutations in certain genes; for example, in melanoma, the expression of YY1 is reduced by NOX3 and RTCB mutations. The above suggests that the impact of YY1 gene mutation is profound, and further experiments are needed to verify it.

We used CPTAC dataset to investigate the underlying molecular process of YY1 protein in six tumor types from the perspective of total protein and phosphorylated protein. In comparison to the normal control group, YY1 total protein expression was shown to be greater in primary tissues of breast, ovarian, colon, and lung adenocarcinoma and lower in primary tissues of clear cell RCC and UCEC. The S118 site of YY1 showed a higher phosphorylation level in breast cancer, colon cancer, and UCEC and LUAD tumor tissues, and lower phosphorylation levels were found in ovarian cancer and clear cell carcinoma. For S247, higher phosphorylation levels were found in colon cancer and UCEC and LUAD tumors, and the tumor tissue of clear cell carcinoma showed lower phosphorylation level. S247 phosphorylation of YY1 is required for the control of neuronal activity, according to Wu's research [42]. The phosphorylation of YY1 at serine 118 and the control of its cleavage during programmed cell death have been identified by Riman et al. [43]. However, we still cannot rule out that the high phosphorylation levels of S247 and S118 of YY1 are a byproduct of dysregulation of tumor cell signaling and have no functional significance in tumor cells. It is necessary to further evaluate the possible function of YY1 phosphorylation in tumorigenesis.

The tumor microenvironment contained a variety of cells. Among them, infiltrating immune cells account for a large proportion. On the one hand, unlike the traditional concept of immune cells as a component of antitumor strategy, immune infiltration in tumor microenvironment reflected the strategy of tumor cells to avoid being killed. Furthermore, in addition to macrophages, almost all types of immune cells, including B cells, CD8^+^ T cells, CD4^+^ T cells, NK cells, and dendritic cells (DC), are present in the tumor microenvironment, and some are involved in the development of cancer. Tumor immuno-infiltrating cells migrate from blood to tumor tissue and play an important role in immune regulation. More and more studies have shown that tumor immuno-infiltrating cells are closely related to immune checkpoint inhibition and prognosis [44–46]. To clarify the relationship between YY1 expression and various immune cells, we first detected the infiltration of 24 kinds of immune cells in 36 tumor types in TCGA database by ssGSEA method and then studied the relationship between YY1 and immune cell infiltration by Spearman analysis method to determine whether YY1 expression is associated with levels of immune infiltration in different cancers. The results showed that YY1 expression level in 12 cancer tissues was significantly negatively correlated with the expression level of most immune cells. YY1 expression level was negatively correlated with B cell infiltration level in 12 kinds of cancer tissues. In addition, the expression level of YY1 was significantly negatively correlated with the infiltration level of CD8^+^ T lymphocytes in 12 kinds of cancer tissues, the infiltration level of neutrophils in 11 kinds of cancer tissues, the infiltration level of macrophages in 8 kinds of cancer tissues, and the infiltration level of dendritic cells in 12 kinds of cancer tissues. These results suggest that YY1 may be a key immunomodulatory target in tumor progression.

We the first time merged the data of YY1-binding protein and YY1 expression-related genes in all cancers in this research and then conducted a suite of enrichment analyses to confirm “viral carcinogenesis,” “microRNAs in cancer,” “covalent chromatin modification,” and “histone modification” of potential impact on cancer etiology and pathogenesis. Recent research has discovered that YY1 is linked to tumor immunity. YY1 was discovered to be highly expressed in various malignancies and to be important in controlling tumor cell drug resistance to cell-mediated immunotherapy, according to Emily et al. They hypothesized that YY1's function in cancer immune resistance was linked to programmed cell death ligand 1 (PD-L1) overexpression in tumor cells, and they discovered multiple signal crosstalk pathways between YY1 and PD-L1 expression regulation [47]. Our study is the first to suggest that YY1 expression is associated to the level of immune invasion of tumor-associated fibroblasts in some tumors, whereas the function of YY1 in tumor immunity and the tumor microenvironment is yet unknown, and the pathogenesis of tumor needs to be further explored.

GSEA analysis was performed to explore the signaling pathways affected by YY1 expression in 36 tumors. Among the results, none of the enrichment results of CHOL, COAD, DLBC, KICH, LUAD, STAD, and ESAD met *P* value <0.05 and FDR *q* value <0.25 condition. GSEA analysis results showed that the expression of YY1 in ACC may be related to hdacs deacetylate histones, hcmv early events, role of lat2 ntal lab on calcium mobilization, initial triggering of complement, and creation of c4 and c2 activators. In BLCA, YY1 expression may be related to the formation of the cornified envelope, cd22-mediated bcr regulation, fcgr activation, scavenging of heme from plasma, and creation of c4 and c2 activators. In BRCA, the expression of YY1 may be related to cd22 mediated bcr regulation, creation of c4 and c2 activators, initial triggering of complement, scavenging of heme from plasma and fcgr activation. YY1 expression in OV may interact with basement membranes, extracellular matrix (ECM) receptor interaction, Wnt signaling, and ribosome and eukaryotic translation of plants. Functional enrichment and protein network analysis can help us study the protein transcription factor network of YY1 in different cancers and then predict its mechanism of action.

Despite the fact that this study used various databases to perform a thorough pan-cancer analysis of YY1, it still has some limitations. To begin, only bioinformatics analytic methodologies and statistical algorithms were used in this work. At the same time, as the analyzed results only comes from the public database, a single data source may lead to certain deviations in our analysis results. Due to the limitation of experimental conditions, specific experimental and clinical studies could not be carried out to prove the possible function of YY1 in generalized tumor. Secondly, unlike in vivo and in vitro research, this study primarily focused on bioinformatics analysis of YY1 expression and patient survival utilizing several databases. More research into the mechanism of YY1 at the cellular and molecular levels will assist to elucidate its function in various cancers. Third, while we identified a connection between YY1 expression and tumor immune cell infiltration, we were unable to determine whether YY1 influences patient survival through immune infiltration. Future prospective studies of YY1 expression and immune cell infiltration in different cancer populations may help to shed more light on the problem's mechanisms.

## 5. Conclusions

In summary, the expression of YY1 was statistically correlated with clinical prognosis, protein phosphorylation, and immune cell infiltration in our initial pan-cancer study. This work used existing data to explore the possible function of YY1 in tumor, generating recommendations for future cancer research.

## Data Availability

The datasets generated and analyzed during the current study are available in the GEO and TCGA repository, [https://www.ncbi.nlm.nih.gov/geo/], and [https://www.cancer.gov/about-nci/organization/ccg/research/structural-genomics/tcga].

## Supplementary Materials

Supplementary 1 Figure S1 The RNA expression of YY1 in normal human tissues. At the RNA level, YY1 was highest in the bone marrow, followed by the thymus and liver. Figure S2 Protein expression of YY1 in normal human tissues. At the protein level, YY1 was highly expressed in urinary bladder, testis, ovary, and placenta tissues. Figure S3 The RNA expression of YY1 in various cancer cell lines. Among various cancer cell lines, U-698 and MOLT-4 cell lines ranked first and second in YY1 expression levels, which were lymphoid derived malignancies, followed by HL-60 and THP-1 cell lines, which were bone marrow derived. Figure S4 The protein expression of YY1 in different human cancer tissues. Among various human cancer tissues, protein expression of YY1 was highest in head and neck tumors, followed by breast cancer. Figure S5 The effects of YY1 mutation on expression. The effects of YY1 mutations in different tumor samples in the TCGA cohort on gene expression.

Supplementary 2 Table S1. Associations between clinicopathologic factors and BLCA patient OS by the use of univariate and multivariate assays. Table S2. Associations between clinicopathologic factors and BLCA patient DSS by the use of univariate and multivariate assays. Table S3. Associations between clinicopathologic factors and BLCA patient PFI by the use of univariate and multivariate assays. Table S4. Associations between clinicopathologic factors and KIRC patient OS by the use of univariate and multivariate assays. Table S5. Associations between clinicopathologic factors and KIRC patient DSS by the use of univariate and multivariate assays. Table S6. Associations between clinicopathologic factors and KIRC patient DSS by the use of univariate and multivariate assays. Table S7. Associations between clinicopathologic factors and MESO patient OS by the use of univariate and multivariate assays. Table S8. Associations between clinicopathologic factors and MESO patient DSS by the use of univariate and multivariate assays. Table S9. Associations between clinicopathologic factors and MESO patient. PFI by the use of univariate and multivariate assays. Table S10. Associations between clinicopathologic factors and OV patient OS by the use of univariate and multivariate assays. Table S11. Associations between clinicopathologic factors and OV patient DSS by the use of univariate and multivariate assays. Table S12. Associations between clinicopathologic factors and OV patient PFI by the use of univariate and multivariate assays.
