# Supplementary figures and images for: A Systematic Pan-Cancer Analysis of YY1 Aberrations and their Relationship with Clinical Outcome, Tumor Microenvironment, and Therapeutic Targets
Source: J Immunol Res. 2022 Jun 24;2022:5826741. doi: 10.1155/2022/5826741 (PMC9250692; doi:10.1155/2022/5826741)

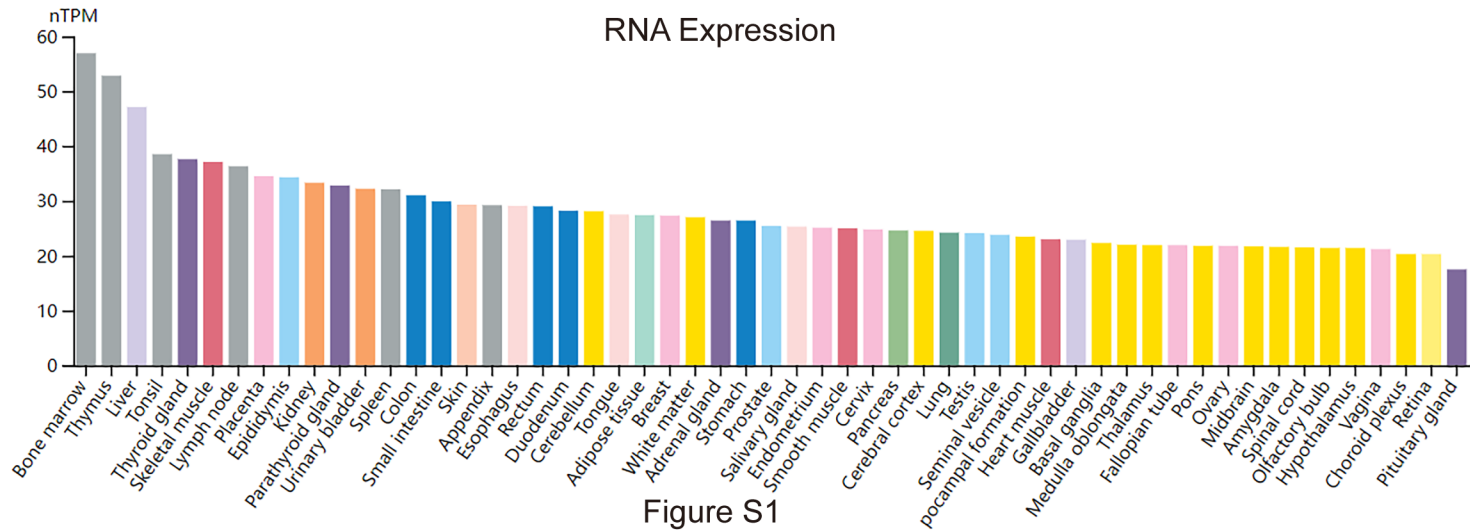

Figure S1  
The RNA expression of YY1 in normal human tissues.

Supplement: Supplementary 1 — Figure S1 The RNA expression of YY1 in normal human tissues. At the RNA level, YY1 was highest in the bone marrow, followed by the thymus and liver. Figure S2 Protein expression of YY1 in normal human tissues. At the protein level, YY1 was highly expressed in urinary bladder, testis, ovary, and placenta tissues. Figure S3 The RNA expression of YY1 in various cancer cell lines. Among various cancer cell lines, U-698 and MOLT-4 cell lines ranked first and second in YY1 expression levels, which were lymphoid derived malignancies, followed by HL-60 and THP-1 cell lines, which were bone marrow derived. Figure S4 The protein expression of YY1 in different human cancer tissues. Among various human cancer tissues, protein expression of YY1 was highest in head and neck tumors, followed by breast cancer. Figure S5 The effects of YY1 mutation on expression. The effects of YY1 mutations in different tumor samples in the TCGA cohort on gene expression. [file 5826741.f1.zip › Figure S1.pdf]

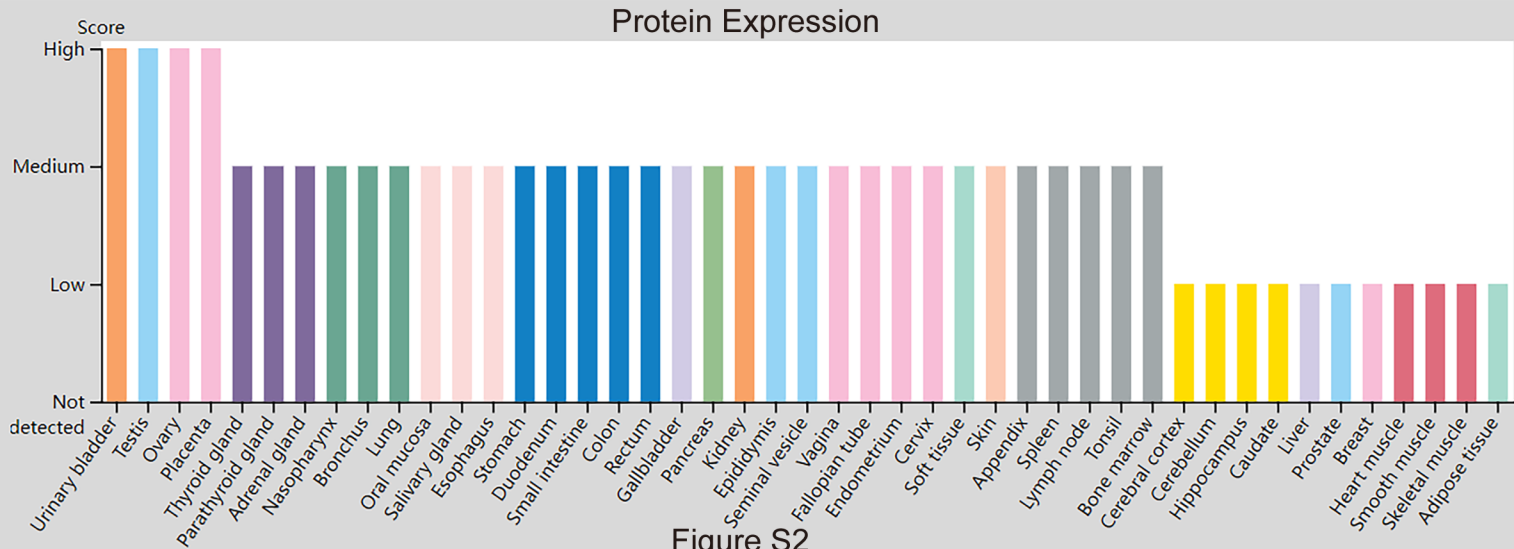

Supplement: Supplementary 1 — Figure S1 The RNA expression of YY1 in normal human tissues. At the RNA level, YY1 was highest in the bone marrow, followed by the thymus and liver. Figure S2 Protein expression of YY1 in normal human tissues. At the protein level, YY1 was highly expressed in urinary bladder, testis, ovary, and placenta tissues. Figure S3 The RNA expression of YY1 in various cancer cell lines. Among various cancer cell lines, U-698 and MOLT-4 cell lines ranked first and second in YY1 expression levels, which were lymphoid derived malignancies, followed by HL-60 and THP-1 cell lines, which were bone marrow derived. Figure S4 The protein expression of YY1 in different human cancer tissues. Among various human cancer tissues, protein expression of YY1 was highest in head and neck tumors, followed by breast cancer. Figure S5 The effects of YY1 mutation on expression. The effects of YY1 mutations in different tumor samples in the TCGA cohort on gene expression. [file 5826741.f1.zip › Figure S2.pdf]

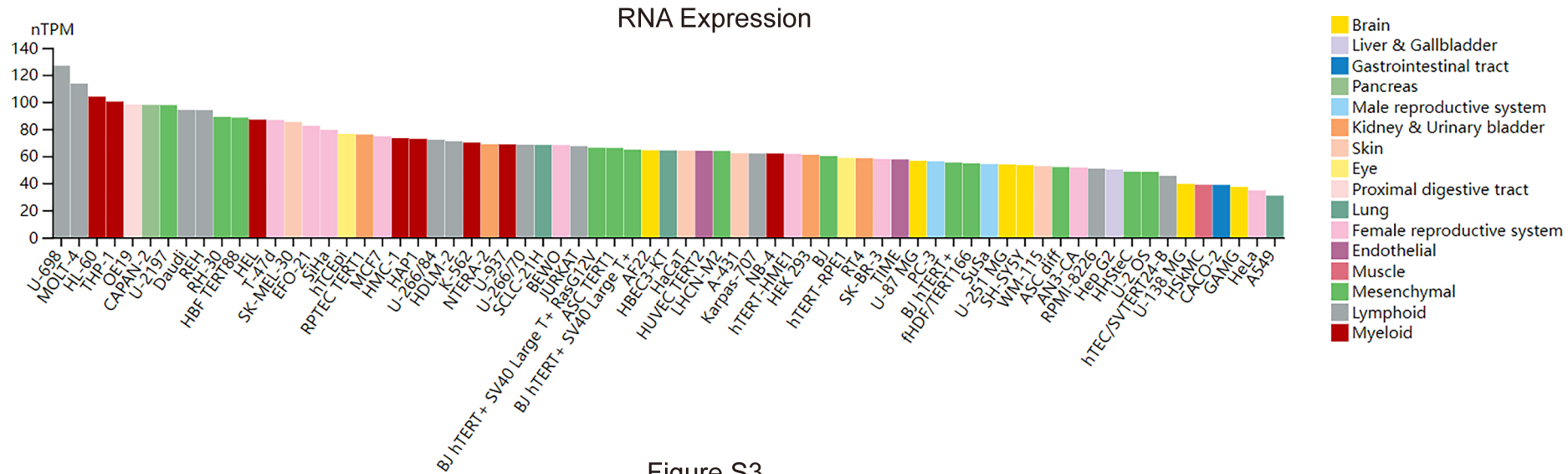

Supplement: Supplementary 1 — Figure S1 The RNA expression of YY1 in normal human tissues. At the RNA level, YY1 was highest in the bone marrow, followed by the thymus and liver. Figure S2 Protein expression of YY1 in normal human tissues. At the protein level, YY1 was highly expressed in urinary bladder, testis, ovary, and placenta tissues. Figure S3 The RNA expression of YY1 in various cancer cell lines. Among various cancer cell lines, U-698 and MOLT-4 cell lines ranked first and second in YY1 expression levels, which were lymphoid derived malignancies, followed by HL-60 and THP-1 cell lines, which were bone marrow derived. Figure S4 The protein expression of YY1 in different human cancer tissues. Among various human cancer tissues, protein expression of YY1 was highest in head and neck tumors, followed by breast cancer. Figure S5 The effects of YY1 mutation on expression. The effects of YY1 mutations in different tumor samples in the TCGA cohort on gene expression. [file 5826741.f1.zip › Figure S3.pdf]

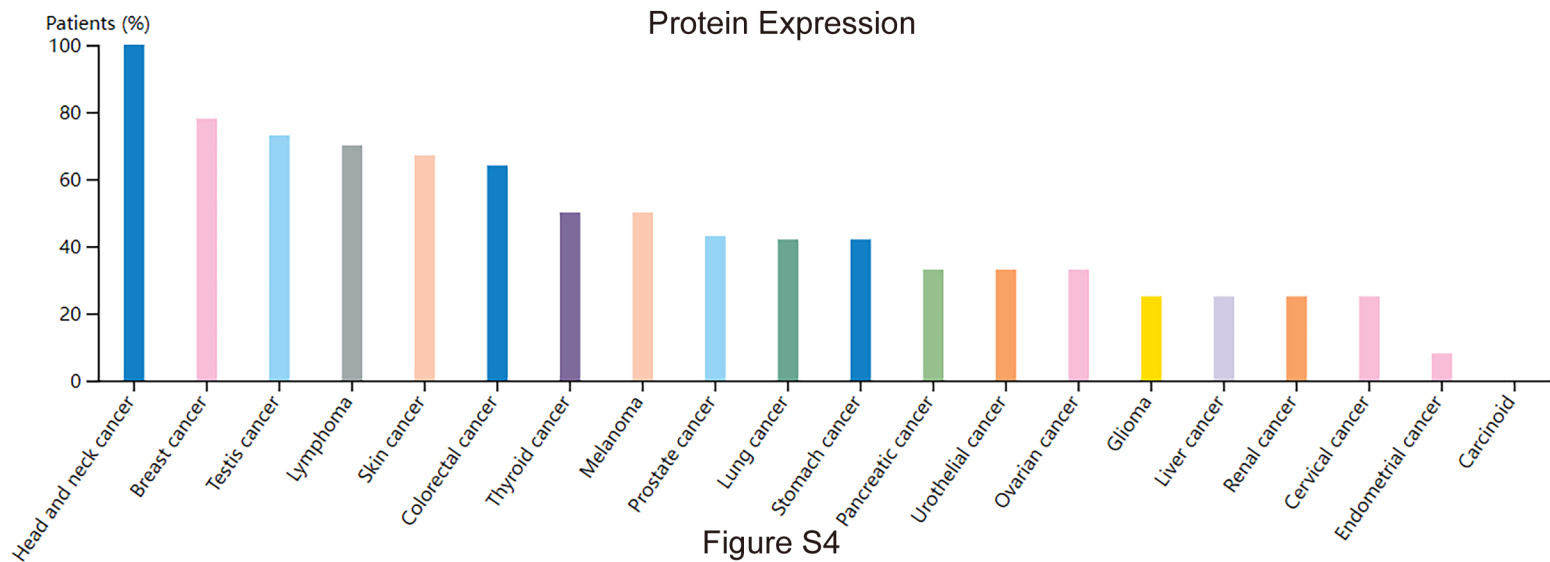

Figure S4  
The protein expression of YY1 in different human cancer tissues.

Supplement: Supplementary 1 — Figure S1 The RNA expression of YY1 in normal human tissues. At the RNA level, YY1 was highest in the bone marrow, followed by the thymus and liver. Figure S2 Protein expression of YY1 in normal human tissues. At the protein level, YY1 was highly expressed in urinary bladder, testis, ovary, and placenta tissues. Figure S3 The RNA expression of YY1 in various cancer cell lines. Among various cancer cell lines, U-698 and MOLT-4 cell lines ranked first and second in YY1 expression levels, which were lymphoid derived malignancies, followed by HL-60 and THP-1 cell lines, which were bone marrow derived. Figure S4 The protein expression of YY1 in different human cancer tissues. Among various human cancer tissues, protein expression of YY1 was highest in head and neck tumors, followed by breast cancer. Figure S5 The effects of YY1 mutation on expression. The effects of YY1 mutations in different tumor samples in the TCGA cohort on gene expression. [file 5826741.f1.zip › Figure S4.pdf]

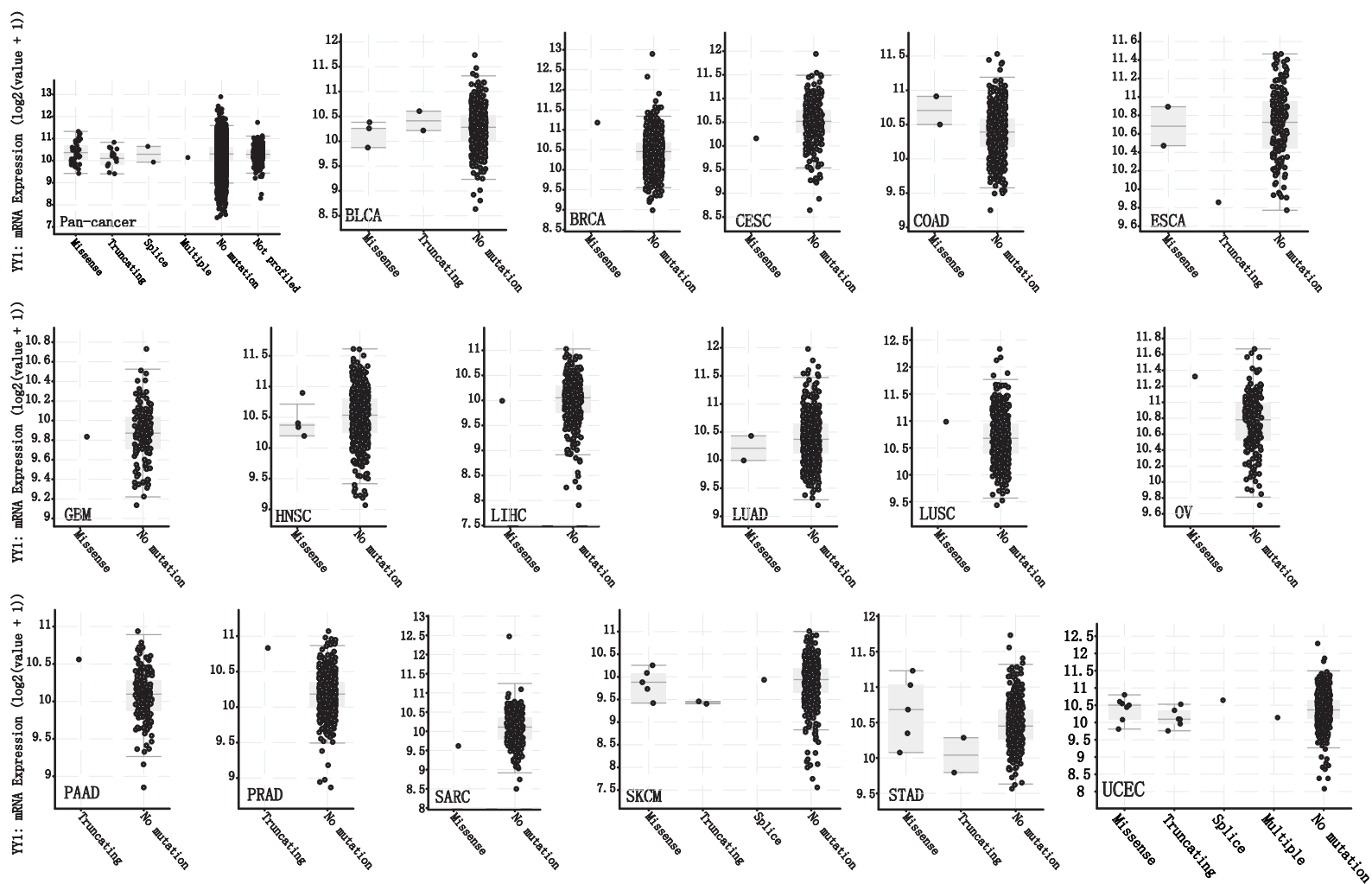

Figure S5

The effect of YY1 mutation on expression

Supplement: Supplementary 1 — Figure S1 The RNA expression of YY1 in normal human tissues. At the RNA level, YY1 was highest in the bone marrow, followed by the thymus and liver. Figure S2 Protein expression of YY1 in normal human tissues. At the protein level, YY1 was highly expressed in urinary bladder, testis, ovary, and placenta tissues. Figure S3 The RNA expression of YY1 in various cancer cell lines. Among various cancer cell lines, U-698 and MOLT-4 cell lines ranked first and second in YY1 expression levels, which were lymphoid derived malignancies, followed by HL-60 and THP-1 cell lines, which were bone marrow derived. Figure S4 The protein expression of YY1 in different human cancer tissues. Among various human cancer tissues, protein expression of YY1 was highest in head and neck tumors, followed by breast cancer. Figure S5 The effects of YY1 mutation on expression. The effects of YY1 mutations in different tumor samples in the TCGA cohort on gene expression. [file 5826741.f1.zip › Figure S5.pdf]
