# Supplementary material for: A Systematic Pan-Cancer Analysis of YY1 Aberrations and their Relationship with Clinical Outcome, Tumor Microenvironment, and Therapeutic Targets
Source: J Immunol Res. 2022 Jun 24;2022:5826741. doi: 10.1155/2022/5826741 (PMC9250692; doi:10.1155/2022/5826741)
Supplement: Supplementary 2 — Table S1. Associations between clinicopathologic factors and BLCA patient OS by the use of univariate and multivariate assays. Table S2. Associations between clinicopathologic factors and BLCA patient DSS by the use of univariate and multivariate assays. Table S3. Associations between clinicopathologic factors and BLCA patient PFI by the use of univariate and multivariate assays. Table S4. Associations between clinicopathologic factors and KIRC patient OS by the use of univariate and multivariate assays. Table S5. Associations between clinicopathologic factors and KIRC patient DSS by the use of univariate and multivariate assays. Table S6. Associations between clinicopathologic factors and KIRC patient DSS by the use of univariate and multivariate assays. Table S7. Associations between clinicopathologic factors and MESO patient OS by the use of univariate and multivariate assays. Table S8. Associations between clinicopathologic factors and MESO patient DSS by the use of univariate and multivariate assays. Table S9. Associations between clinicopathologic factors and MESO patient. PFI by the use of univariate and multivariate assays. Table S10. Associations between clinicopathologic factors and OV patient OS by the use of univariate and multivariate assays. Table S11. Associations between clinicopathologic factors and OV patient DSS by the use of univariate and multivariate assays. Table S12. Associations between clinicopathologic factors and OV patient PFI by the use of univariate and multivariate assays. [file 5826741.f2.zip › Table S2 BLCA DSS.docx]

**Table S2. Association between clinicopathologic characteristics and BLCA patient DSS through univariate and multivariate analysis with Cox regression survival model**

| Characteristics | Total(N) | Univariate analysis | |  | Multivariate analysis | |
| --- | --- | --- | --- | --- | --- | --- |
|  |  | Hazard ratio (95% CI) | P value |  | Hazard ratio (95% CI) | P value |
| T stage | 366 |  |  |  |  |  |
| T1&T2 | 124 | Reference |  |  |  |  |
| T3&T4 | 242 | 2.248 (1.432-3.527) | **<0.001** |  | 0.966 (0.217-4.295) | 0.963 |
| N stage | 357 |  |  |  |  |  |
| N0&N1 | 278 | Reference |  |  |  |  |
| N2&N3 | 79 | 2.784 (1.894-4.091) | **<0.001** |  | 1.882 (0.786-4.509) | 0.156 |
| M stage | 208 |  |  |  |  |  |
| M0 | 197 | Reference |  |  |  |  |
| M1 | 11 | 4.205 (1.889-9.359) | **<0.001** |  | 0.776 (0.163-3.682) | 0.749 |
| Pathologic stage | 397 |  |  |  |  |  |
| Stage I&Stage II | 134 | Reference |  |  |  |  |
| Stage III&Stage IV | 263 | 2.544 (1.617-4.001) | **<0.001** |  | 2.495 (0.379-16.433) | 0.342 |
| Radiation therapy | 376 |  |  |  |  |  |
| No | 355 | Reference |  |  |  |  |
| Yes | 21 | 1.014 (0.446-2.305) | 0.974 |  |  |  |
| Gender | 399 |  |  |  |  |  |
| Female | 103 | Reference |  |  |  |  |
| Male | 296 | 0.849 (0.576-1.251) | 0.408 |  |  |  |
| Race | 382 |  |  |  |  |  |
| Asian | 43 | Reference |  |  |  |  |
| Black or African American&White | 339 | 2.107 (0.858-5.176) | 0.104 |  |  |  |
| Age | 399 |  |  |  |  |  |
| <=70 | 226 | Reference |  |  |  |  |
| >70 | 173 | 1.026 (0.718-1.466) | 0.888 |  |  |  |
| Weight | 359 |  |  |  |  |  |
| <=80 | 198 | Reference |  |  |  |  |
| >80 | 161 | 0.898 (0.615-1.311) | 0.578 |  |  |  |
| Height | 354 |  |  |  |  |  |
| <=170 | 153 | Reference |  |  |  |  |
| >170 | 201 | 1.184 (0.802-1.750) | 0.395 |  |  |  |
| BMI | 353 |  |  |  |  |  |
| <=25 | 147 | Reference |  |  |  |  |
| >25 | 206 | 1.036 (0.698-1.538) | 0.859 |  |  |  |
| Histologic grade | 396 |  |  |  |  |  |
| High Grade | 375 | Reference |  |  |  |  |
| Low Grade | 21 | 0.461 (0.114-1.870) | 0.278 |  |  |  |
| Subtype | 394 |  |  |  |  |  |
| Non-Papillary | 265 | Reference |  |  |  |  |
| Papillary | 129 | 0.577 (0.371-0.897) | **0.015** |  | 1.566 (0.631-3.888) | 0.334 |
| Lymphovascular invasion | 274 |  |  |  |  |  |
| No | 127 | Reference |  |  |  |  |
| Yes | 147 | 3.048 (1.894-4.905) | **<0.001** |  | 3.048 (1.150-8.080) | **0.025** |
| Smoker | 387 |  |  |  |  |  |
| No | 105 | Reference |  |  |  |  |
| Yes | 282 | 1.305 (0.860-1.980) | 0.211 |  |  |  |
| YY1 | 399 |  |  |  |  |  |
| Low | 201 | Reference |  |  |  |  |
| High | 198 | 1.249 (0.877-1.778) | 0.219 |  |  |  |
